# Development and evaluation of RFID-integrated endoscopic clips for laparoscopic surgery marking

**DOI:** 10.1371/journal.pone.0302737

**Published:** 2024-05-02

**Authors:** Hwan Yi Joo, Cho Rong Park, Seokyoung Ahn, Chang In Choi

**Affiliations:** 1 School of Mechanical Engineering, Pusan National University, Busan, South Korea; 2 Department of Surgery, Biomedical Research Institute, Pusan National University Hospital, Pusan National University College of Medicine, Busan, South Korea; University of Vigo, SPAIN

## Abstract

**Background:**

As advancements in surgical instruments and techniques continue to evolve, minimally invasive surgery has become increasingly preferred as a means of reducing patient pain and recovery time. However, one major challenge in performing minimally invasive surgery for early gastrointestinal cancer is accurately identifying the location of the lesion. This is particularly difficult when the lesion is confined to the lumen of the intestine and cannot be visually confirmed from the outside during surgery. In such cases, surgeons must rely on CT or endoscopic imaging to locate the lesion. However, if the lesion is difficult to identify with these images or if the surgeon has less experience, it can be challenging to determine its precise location. This can result in an excessive resection margin, deviating from the goal of minimally invasive surgery. To address this challenge, researchers have been studying the development of a marker for identifying the lesion using a radio-frequency identification (RFID) system. One proposed method for clinical application of this detection system is to attach an RFID tag to an endoscopic hemostatic clip and fix it to the intended position, providing a stable marker for the inner wall of the organ. This approach has the potential to improve the accuracy and effectiveness of minimally invasive surgery for early gastrointestinal cancer.

**Methods:**

In the development of a marker for identifying gastrointestinal lesions using a radio-frequency identification (RFID) system, the shape of the clip and suitable materials for attaching the RFID tag were determined through finite element method (FEM) analysis. A prototype of the clip was then fabricated and ex-vivo experiments were conducted using porcine intestine to evaluate the stability of the clip in relation to its position. To further evaluate the performance of the RFID-integrated clip in vivo, the clip was placed in the gastric wall of the stomach of anesthetized porcine using an endoscopic instrument. The clip was then detected using a RFID detector designed for laparoscopic approach. And later, the accuracy of detection was confirmed by incising the lesion.

**Results:**

The design and fabrication of a clip with varying thicknesses using STS316 and STS304 stainless steel were accomplished using the results of finite element method analysis. The stability of the clip was evaluated through ex-vivo experiments, showing it to be a viable option. In-vivo experiments were performed on anesthetized porcine, in which the RFID-integrated clip was placed in the gastric wall and detected using a custom-made RFID detector. The resection margin, measured at about 30 mm from the detector position, was accomplished with low error. These findings indicate the feasibility and efficacy of using an RFID-integrated clip as a marker in minimally invasive surgery for the identification of gastrointestinal lesions.

**Conclusions:**

The study evaluated the feasibility of using stainless steel clips for lesion detection in endoscopic surgery using computer-aided engineering analysis and ex-vivo experimentation. Results showed that STS304 was suitable for use while STS316L was not. The ex-vivo experiments revealed that the clip holding force and tissue retention length varied depending on the location of attachment. In-vivo experiments confirmed the accuracy and usefulness of the RFID lesion detection system. However, challenges remain for its use in clinical field, such as ensuring the stability of the clip and the safe attachment of the RFID tag, which requires further research for commercialization.

## Introduction

In cases of early cancer or small benign tumors with endophytic growth, it can be challenging for laparoscopic surgery to accurately identify the exact location of the lesion, as these types of lesions are not visible on the serosal surface of the organ. Surgeons may have to rely on imaging tests or their own experience to predict the potential location of the tumor. This can lead to extensive resection, which contradicts the goal of minimally invasive surgery [1–4].

To address this issue, various studies have been conducted to develop methods for easily detecting the location of lesions in laparoscopic surgery. Hyung et al. proposed a method of confirming the position of a metal clip marked near the lesion using laparoscopic ultrasonography [5], and Kim et al. used portable abdominal radiography to confirm the clip location [6]. While these methods can provide high accuracy in finding the marked location, they require specialized equipment and expertise, and the results may vary depending on the experience and skill of the surgeon interpreting the image data. Furthermore, Lee et al. found that using radiographic images can prolong the overall operation time [7].

To overcome these limitations, Joo et al. suggested a method of detecting the lesion using a radio-frequency identification (RFID) system [8]. This approach is easy and simple, as it does not require complicated operations or expertise. The RFID system allows anyone to easily identify the location of the lesion by detecting the pre-attached marker when a detector is brought close by.

In previous research, it was confirmed that the radio-frequency identification (RFID) markers were correctly activated, and the accuracy of lesion detection was sufficient for clinical application [8]. To further evaluate the feasibility of the RFID detection system under actual surgical conditions, it is necessary to conduct clinical experiments using RFID markers attached to the site that needs to be positioned.

To achieve this, a cylindrical RFID tag was selected as the marker, and an attachment mechanism was proposed. An endoscopic clip was fabricated that could be attached to the inner wall of the intestine using an endoscopic instrument. The clip-type fixing method was used, considering the characteristics of the RFID tag and the conditions of the application area. To ensure the availability of the clip, FEM analysis was performed by diversifying the materials and thickness conditions of the clip, and deformation of the clip was analyzed. The results of the analysis showed that the performance of the instrument and the stability of the clip are feasible. The optimal conditions for clinical use were further validated through experiments in which prototype clips were attached to a porcine intestine.

## Materials and methods

### Clip design

**Fig 1:**
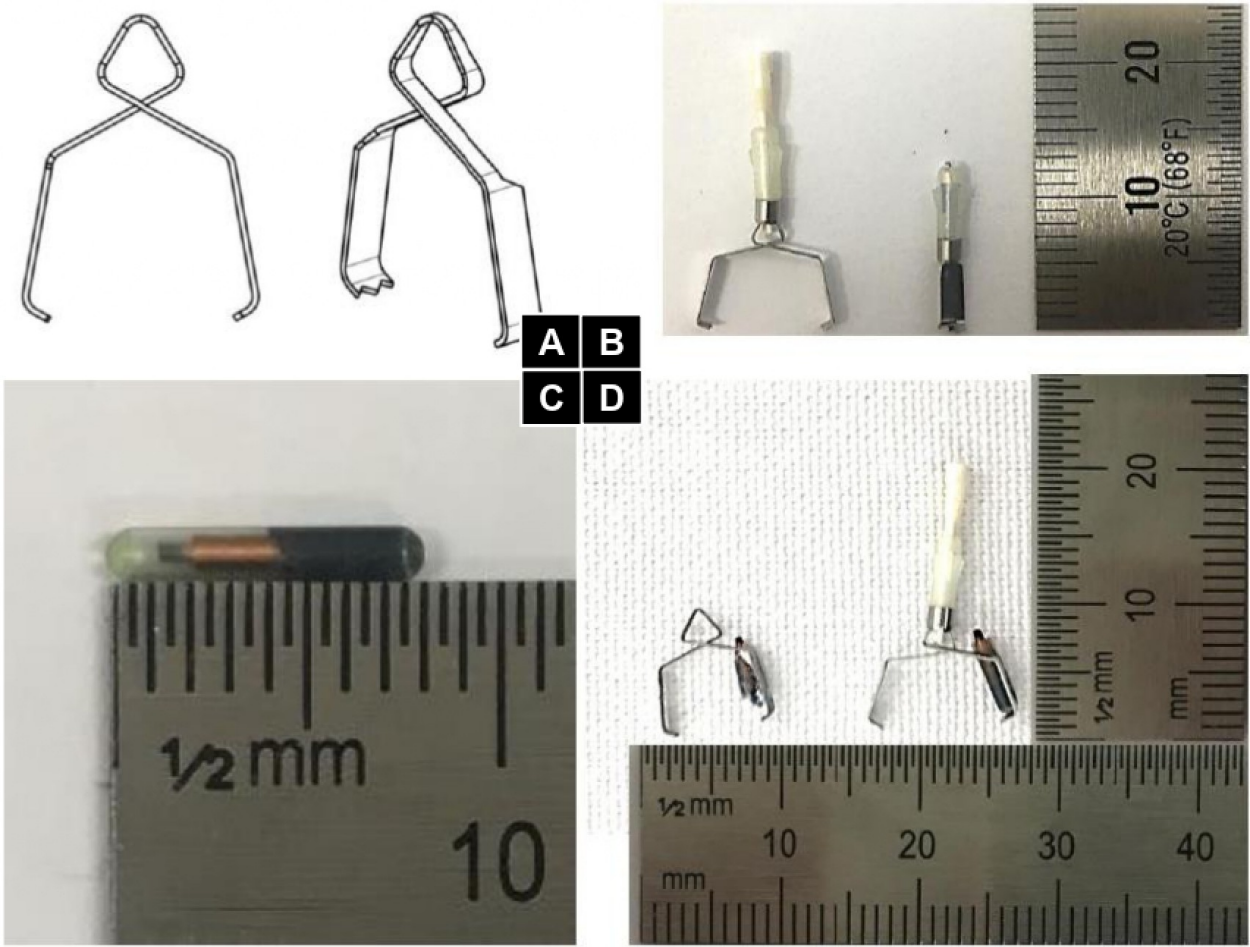


**Fig 2:**
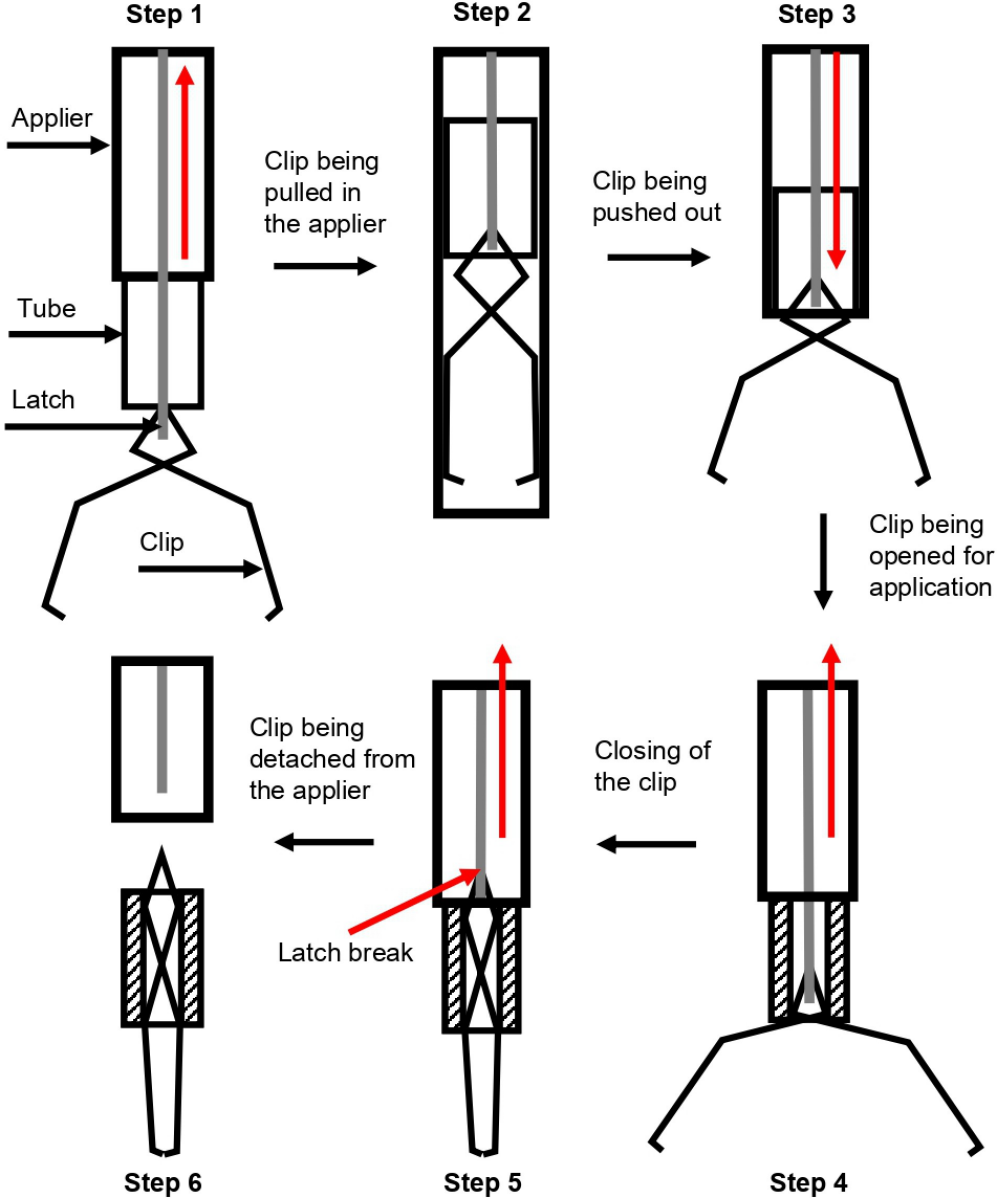


A new method for attaching a radio-frequency identification (RFID) tag (READELL EM4305, Xinyetong, China) to an endoscopic clip was proposed as a clinical solution for lesion detection during minimally invasive surgery. The design of the clip was compatible with the commonly used endoscopic clip fixing applier (EZ-Clip, OLYMPUS, Japan) and was optimized for use in laparoscopic procedures. The design of the clip and its function when housed in the applier are illustrated in Figs 1A and 2 respectively, highlighting the importance of the clip’s elastic restoration characteristics in its performance.

### Materials

The conditions for selecting the materials used to fabricate the clip are as follows:

1. Biocompatibility: The clip should be made of materials that are safe for use in the human body and do not cause additional injury or infection.
2. Corrosion resistance: The clip should be able to withstand exposure to strong acids such as gastric juice as it will be used to mark the location of tumors within the intestine.
3. Non-magnetic: The clip should be made of materials that do not affect the electromagnetic field formed by the RFID tag and antenna coil.

Based on these criteria, STS304 and STS316 types of stainless steel were identified as suitable candidate materials for the clip.

The RFID component is designed by using the EM4305 chipset (EM4305, Xinyetong, China), which operates on the ISO11784/5 protocol. The RFID capsule designed for this purpose is 1.5 mm in diameter and 7.5 mm in length. For the reader, we employed the EM4100 module. The accompanying coil was crafted in a cylindrical form, measuring 8 mm in diameter and 15 mm in height, with a configuration set to 3.6 μH.

### Structural analysis by CAE software

In a previous experiment, the ability of the clip to retain its shape and the suitability of the selected materials were investigated through finite element method (FEM) analysis using a computer-aided engineering (CAE) program (ANSYS Workbench ver19.1). The ability of the clip to restore its shape after being deformed is crucial for its ability to attach around a lesion. To evaluate this, the shape deformation of the clip was analyzed while applying and removing force. For the analysis of nonlinear material, stress-strain curve information was input under the condition of multilinear model.

In the preliminary verification process, it was found that the clip made of 316 type stainless steel was not able to restore its shape after deformation. Therefore, this material was deemed inappropriate for use. Subsequently, only the 304 types of stainless-steel materials were analyzed. The selected material was STS304, which has a basic composition of 304 type stainless steel, and STS304L, which has improved corrosion resistance by lowering the ratio of carbon (C). The thickness of the clip was set to 0.15 mm and 0.2 mm.

### Experiment using a prototype clip

**Fig 3:**
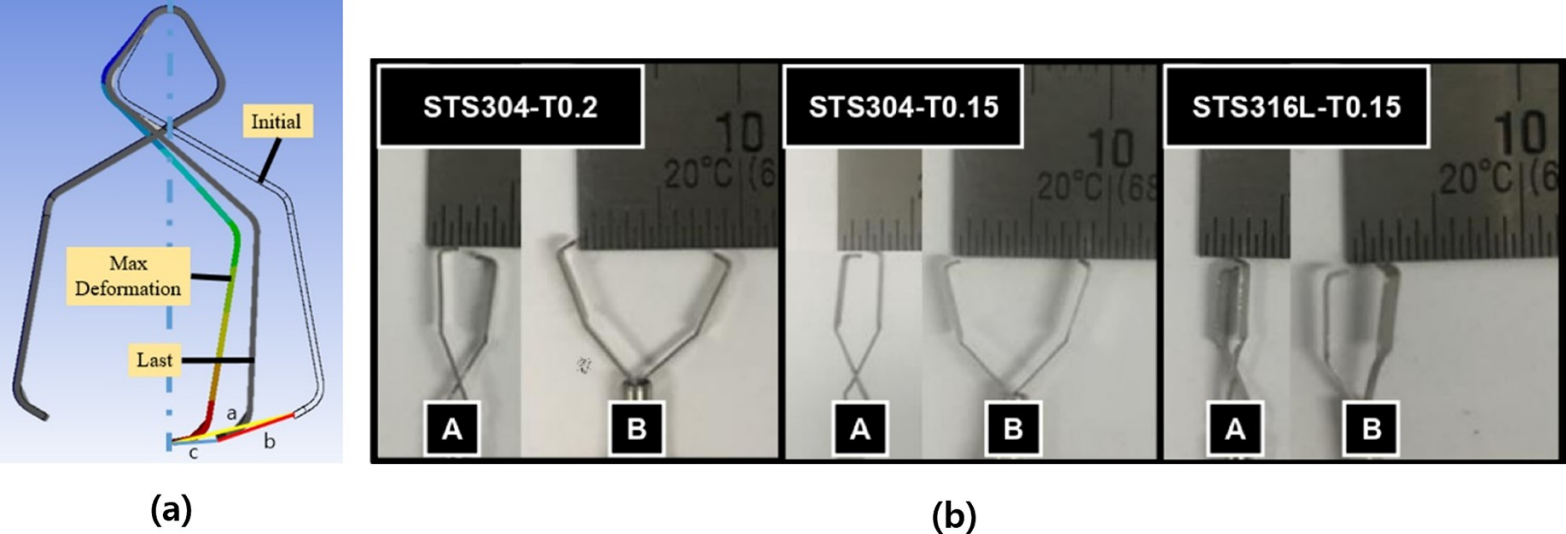


To evaluate the performance of the proposed clip design, three prototype clips were prepared: a clip of STS304 with a thickness of 0.2 mm (STS304-T0.2), a clip of STS304 with a thickness of 0.15 mm (STS304-T0.15), and a clip of STS316L with a thickness of 0.15 mm (STS316L-T0.15). Using these prototype clips, the steps 1 to 4 of Fig 2 were repeated, and the opening length of the clip was measured as depicted in Fig 3. The results of the analysis using the program and the deformation results of the actual clips were then compared.

### Ex-vivo experiment

**Fig 4:**
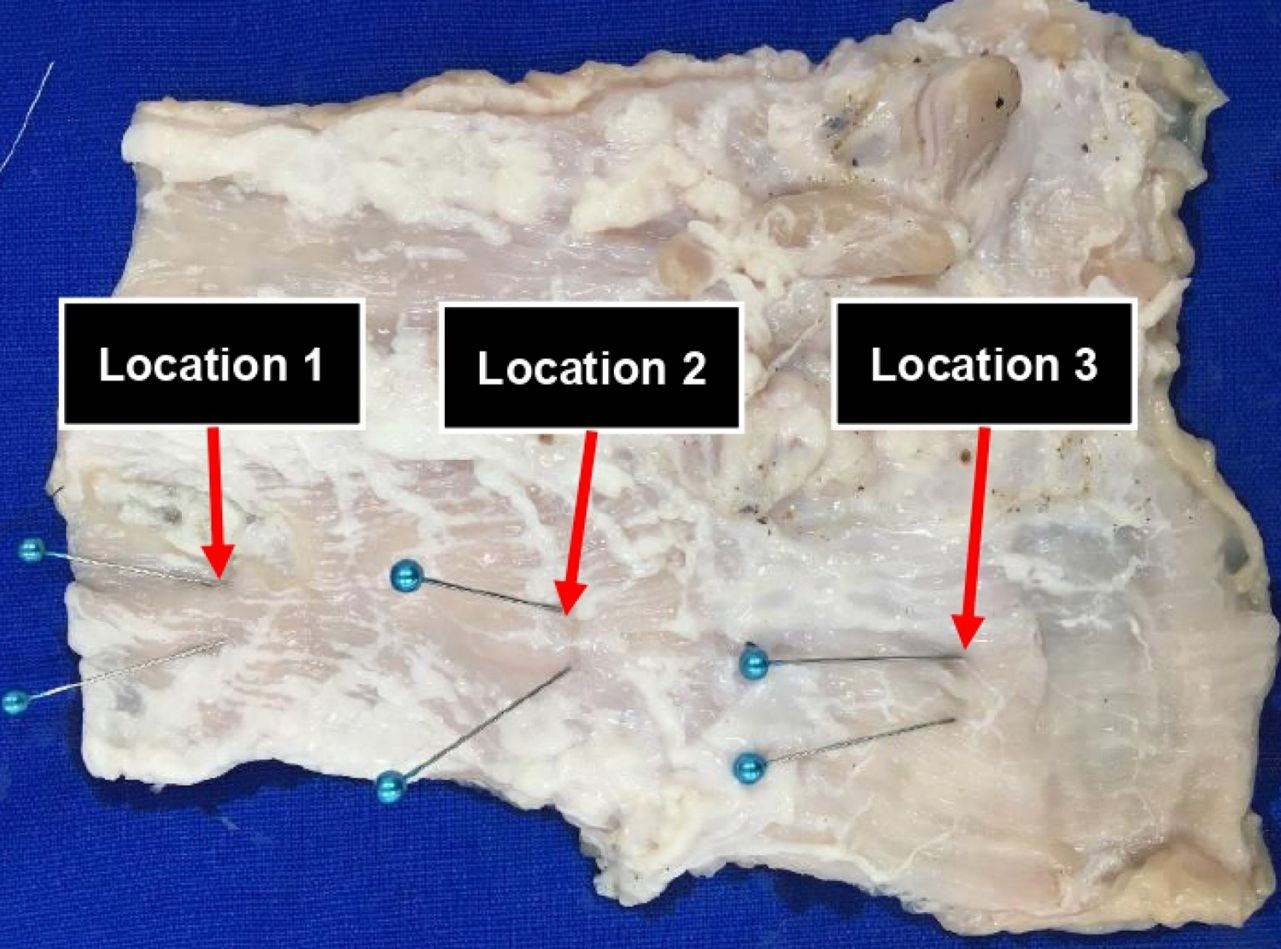


**Fig 5:**
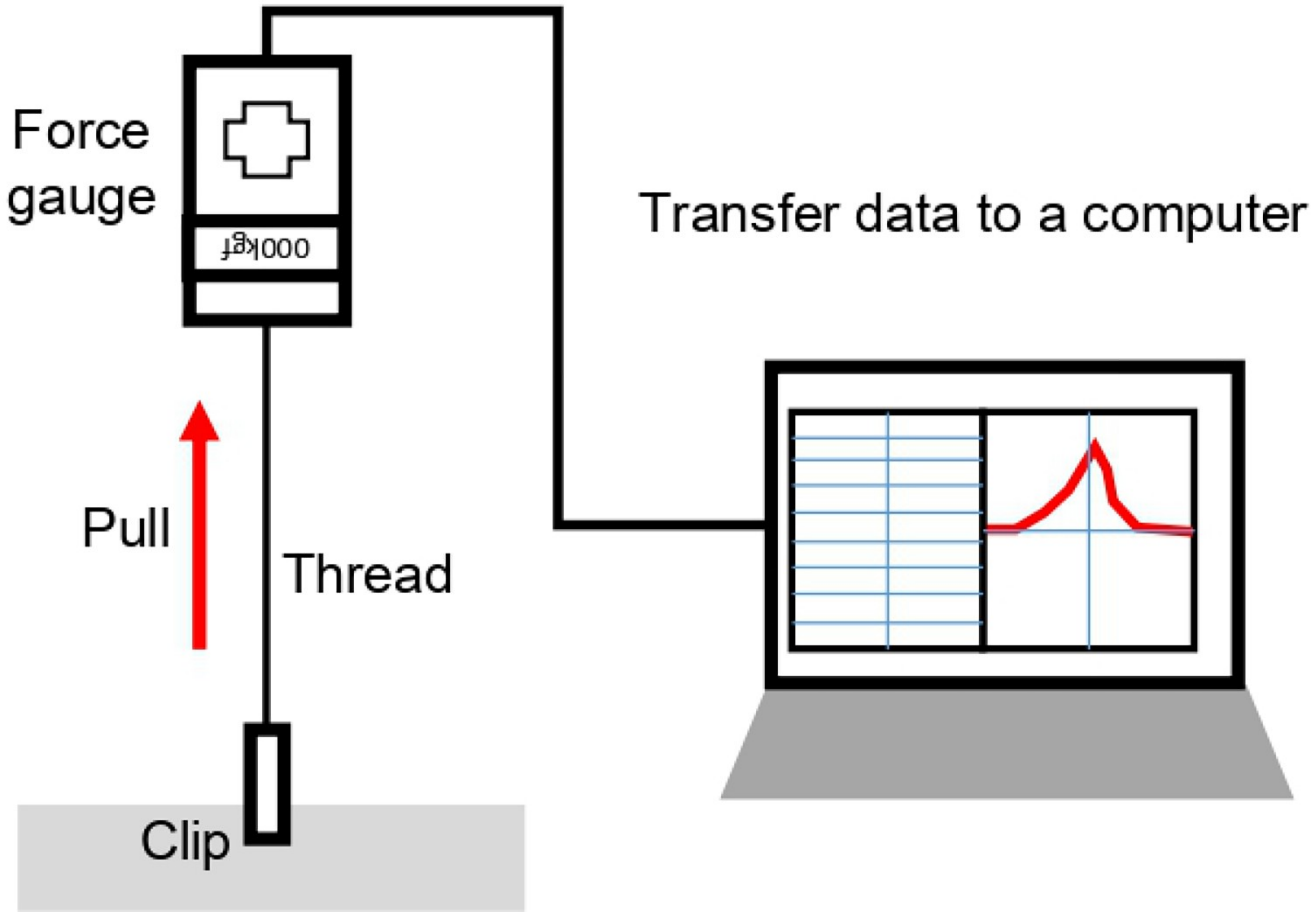


After the prototype of the clip was produced, an experiment was conducted to confirm the attachment and stability of the clip by attaching it to a porcine intestine. A 30 cm segment of porcine colon was used for the ex-vivo experiment. The prototype clips were attached to three different locations in the incised colon (Fig 4). After attaching the prototype clip, the force required to detach the clip from the tissue was measured using a force gauge attached to the clip with thread a shown in Fig 5. After separating the clip, the distance between the pins displayed on both ends of the clip and the thickness of the clip-attached location were also measured.

### In-vivo experiment

The feasibility and lesion detection capability of the radio-frequency identification (RFID) tag-integrated endoscopic clip were evaluated through in-vivo experiments on anesthetized porcine. The administration of general anesthesia was carried out on a porcine weighing 40 kilograms while in a supine position. Deep sedation and pain relief were achieved using 4mg/kg of Rompun (xylazine) and 3mg/kg of Alfaxan (alfaxalone). Intravenous injection of KCL was utilized for euthanasia following the experiment. The animal protocol used in this study has been reviewed by the Pusan National University-Institutional Animal Care and Use Committee (PNU-IACUC) on their ethical procedures and scientific care, and it has been approved (Approval Number, 2017–046).

The clip was attached to the proximal porcine stomach through an endoscopic instrument, and a detector was inserted into the abdominal cavity through a laparoscopic port to locate the clip. Subsequently, the location identified by the detector was marked, and the porcine stomach was resected with the proximal and distal margins set at 30 mm from the mark. The presence of the clip for lesion detection was confirmed within the incised stomach. While the optimal resection margin for achieving oncological safety remains a topic of ongoing debate, in this study, we aimed to confirm the exact location of the lesion with a 30 mm margin according to the surgeon’s intention.

## Results

### FEM analysis

The restored length of the clip opening was determined by subtracting the final deformation from the maximum deformation of the jaw, or the end of the clip, while applying and removing force. The restoration rate was calculated as the ratio of the maximum deformation to the restoration length. The results of this analysis are presented in Table 1.

**Table 1:** Maximum deformation and restoration length by CAE analysis.

Material	Thickness (mm)	Maximum Force (N)	Maximum Deformation (mm)	Last Deformation (mm)	Restoration Length (mm)	Restoration Rate
STS304	0.15	0.085	3.10	2.02	1.08	34.84%
STS304	0.20	0.156	3.07	2.24	0.83	26.97%
STS304L	0.15	0.071	3.05	2.16	0.89	29.23%

When comparing the restoration length, the 0.15mm thick STS304 clip (STS304-T0.15) exhibited the longest restoration length and the highest restoration rate. However, it should be noted that the STS304-T0.2 clip required the largest maximum force for deformation. Among clips of similar thickness, it is anticipated that a clip made of STS304 would have a superior restoration rate compared to one made of STS304L, making STS304 a more suitable material for the clip’s function. When comparing the STS304-T0.15 and STS304-T0.2, the STS304-T0.15 had a relatively higher restoration rate compared to the STS304-T0.2, but the latter required a greater force to be applied for deformation. It is anticipated that the clipping force of the clip grasping the tissue would be stronger and the stability would be higher with the STS304-T0.2 clip.

### Experiment using the prototype clips

The results of the measurement of the opening length after deformation using the prototype clips are shown in Table 2. The longer the opening length between the jaws at both ends of the clip, the more tissue can be grasped, making the clip more stable when fixed. On the other hand, if the opening length is short, it can be easily detached from tissue or fail to apply the clips due to the small, grasped range. In order to apply the clip to tissue, it should be spread at least 40 degrees, with a more favorable spread of over 90 degrees. The minimum opening length for a minimum angular spread is at least about 7mm.

**Table 2:** Opening length of the prototype clips used by applier.

	Opening length(mm)		
Number of Experiment	STS304-T0.2	STS304-T0.15	STS316L-T0.15
1	8	8	2
2	7	6	5
3	5	7	4
4	7	8	2
5	7.5	8	3
Max	8	8	5
Min	5	6	2
Average	6.9±1.14	7.4±0.89	3.2±1.30

The average opening length of the STS304-T0.2 clips was 6.9±1.14 mm, with a maximum length of 8 mm and a minimum length of 5 mm. The average opening length of the STS304-T0.15 clips was 7.4±0.89 mm, with a maximum length of 8 mm and a minimum length of 6 mm. The average opening length of the STS316L-T0.15 clips was 3.2±1.30 mm, with a maximum length of 5 mm and a minimum length of 2 mm. The STS304-T0.15 clips showed the smallest deviation and similar results compared to the CAE results and operated stably. On the other hand, the STS316L clips had an average opening length of 3.2 mm, which was less than the minimum required length, making them inappropriate for attaching to tissue. As a result, the STS304-T0.15 and STS304-T0.2 clips were fabricated for the ex-vivo experiment.

### Ex-vivo experiment

#### Location 1

**Fig 6:**
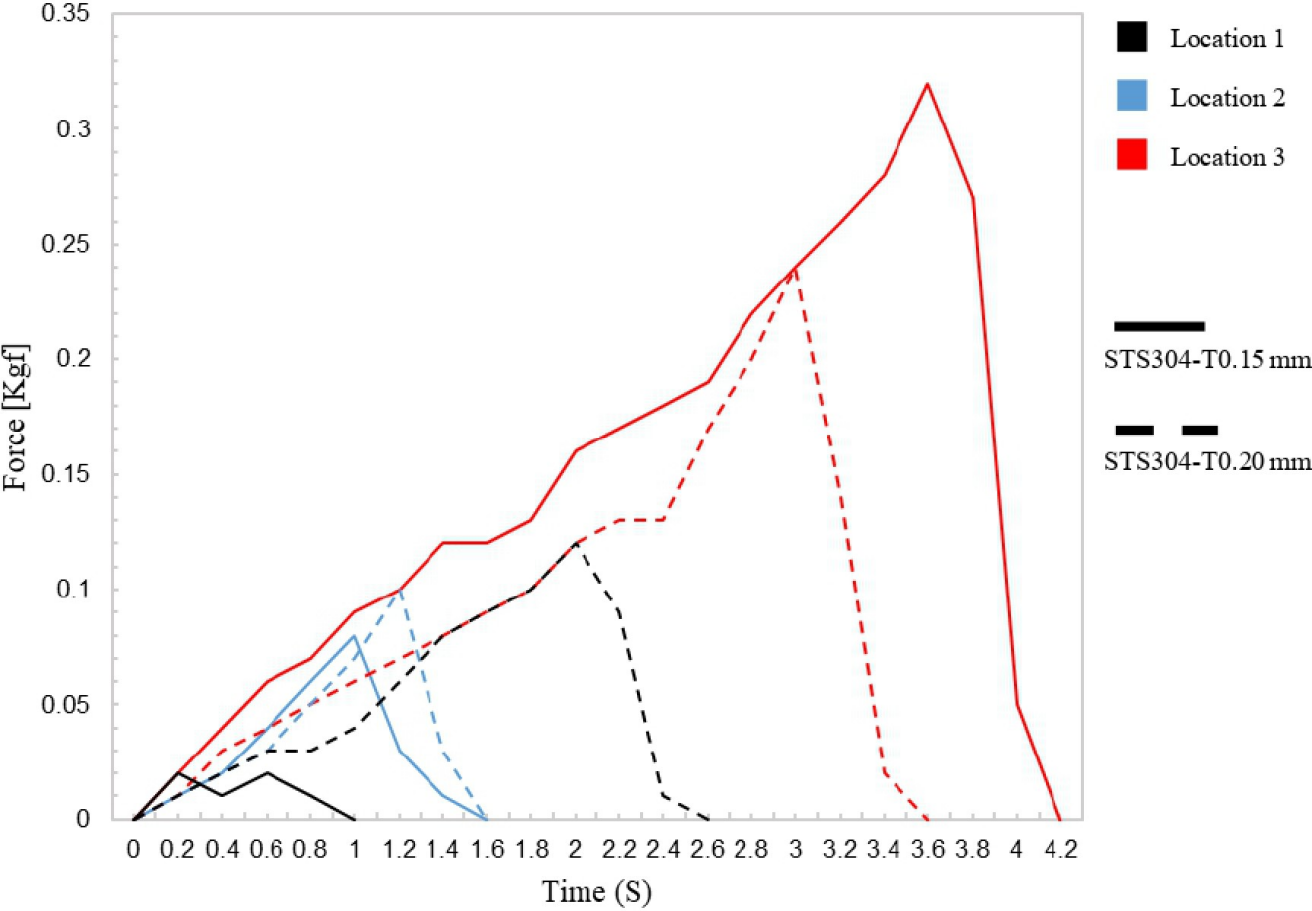


In the ex-vivo experiments, the prototype clips were attached to the porcine intestine and the stability of the clips was evaluated by measuring the thickness of the location where the clips were attached and the force and time required to attach and detach the clips. The results showed that the STS304-T0.15 clips required a stronger force and longer time to detach compared to the STS304-T0.2 clips (Fig 6). Specifically, the maximum force required for the STS304-T0.15 clips was 0.02 kgf, while the STS304-T0.2 clips required 0.12 kgf, a difference of approximately 6 times. The required time was approximately 1 second for the STS304-T0.15 clips and 2.6 seconds for the STS304-T0.2 clips, a difference of more than 2 times. These results indicate that the STS304-T0.15 clips may provide a more stable attachment to the tissue compared to the STS304-T0.2 clips.

#### Location 2

The results of the ex-vivo experiments conducted at location 2, which had a relatively hard tissue texture compared to other locations, are shown in blue in Fig 6. The time and force required to attach and detach the STS304-T0.15 and STS304-T0.2 clips were measured. The results indicate that while the STS304-T0.2 clips required a slightly higher force to detach, the difference was not significant. The maximum force required to detach the STS304-T0.15 clips was 0.08 kgf, while the maximum force required for the STS304-T0.2 clips was 0.1 kgf. The time required for detachment was similar for both types of clips.

#### Location 3

The ex-vivo experiment revealed that the STS304-T0.15 clip required more force and time for separation when applied to the softest location (location 3) compared to the other locations. The maximum force during separation was recorded as 0.32 kgf for the STS304-T0.15 clip and 0.24 kgf for the STS304-T0.2 clip at location 3. These findings are illustrated in the red line of Fig 6.

#### Tissue retention length

The results of measuring the tissue retention length after separating the clips at each location are presented in Table 3. The results indicate that the STS304-T0.2 clip retained a relatively longer range of tissue than the STS304-T0.15 clip at the same position. The tissue retention length was relatively shorter in areas with thicker tissue, such as location 2. Conversely, thinner areas, such as location 1 and location 3, had longer tissue retention length. When comparing the characteristics of the clip-attached locations, the texture was smooth in the order of location 3, location 1, and location 2. Location 2 had a hard texture with little deformation when pressed. Based on this, it can be inferred that clips can retain a larger range of tissue in areas with a soft texture. The average tissue retention length was 8.67 mm for the STS304-T0.15 clip and 10.67 mm for the STS304-T0.2 clip, indicating that the STS304-T0.2 clip is able to retain a longer length of tissue.

**Table 3:** Measured tissue retention length by the clips.

Location	Texture	Thickness	Tissue retention length (mm)	
			STS304-T0.15	STS304-T0.2
1	Smooth	1.5	9	11
2	Hard	5.0	5	8
3	Most Smooth	1.5	12	13
Average			8.67	10.67

### In-vivo experiment

**Fig 7:**
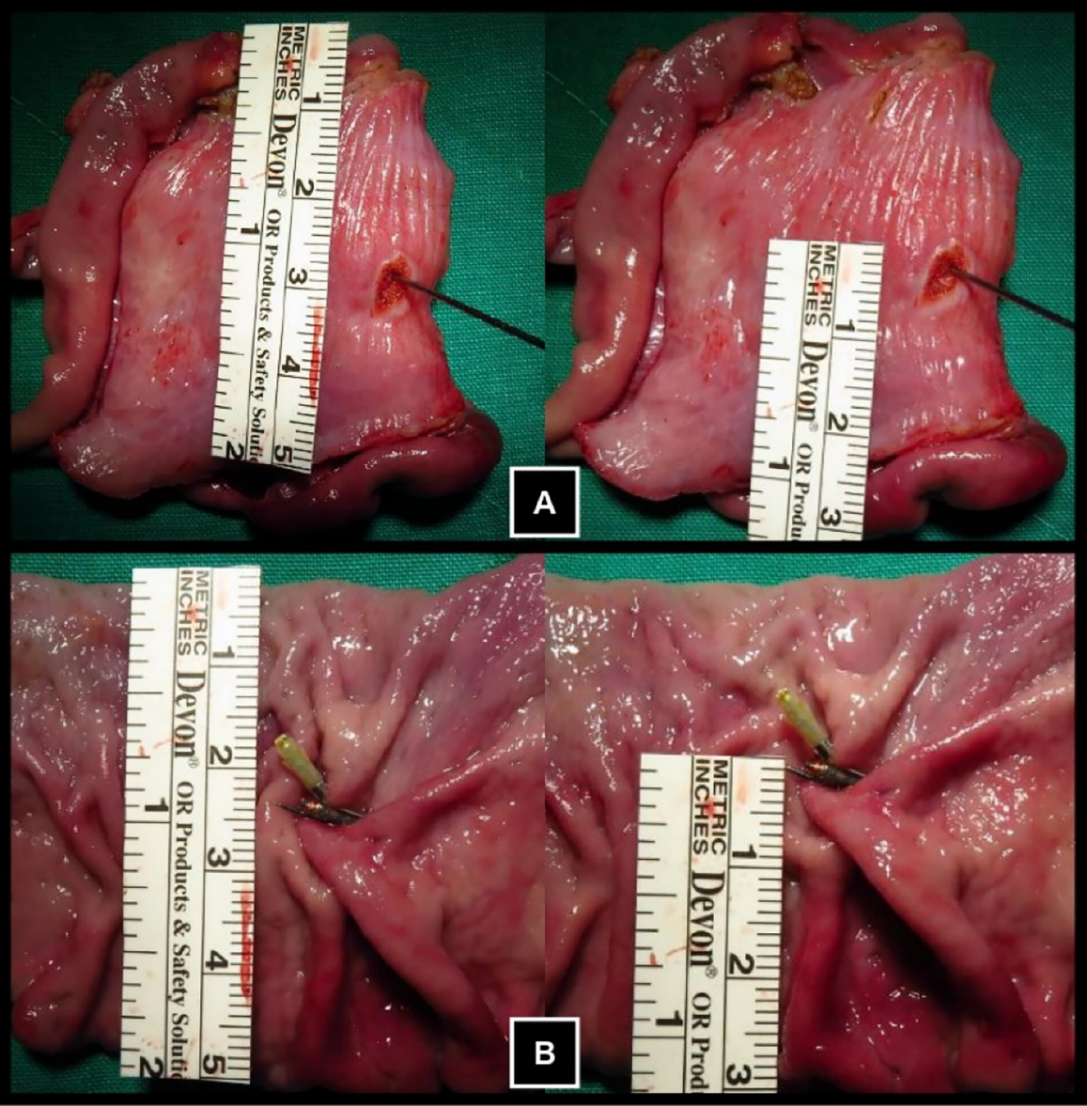


The prototype clip used in the experiment was fabricated from STS304 stainless steel with a thickness of 0.15 mm, as shown in Fig 1B. The RFID tag employed for lesion localization was a cylindrical tag, depicted in Fig 1C. The RFID tag was adhered to the clip using a silicone adhesive and coated with a layer of urethane to protect against strong acid. The RFID tag-integrated endoscopic clip was applied to the proximal portion of the porcine stomach and its location was confirmed using an RFID detector. The results indicated that the RFID lesion detection system functioned as intended. After identifying the clip’s location through the detector and marking the area, the proximal and distal resection margins were set to 30 mm from the marked position. The porcine stomach was then resected along the marked resection margins and it was confirmed that the clip was located within the resected tissue. The clip was confirmed at approximately 30 mm from the incision margin, as the surgeon’s intention. The results indicated a high level of accuracy in the detected location, as the location of the clip and the outer marking location were nearly identical. The resected specimen is presented in Fig 7.

## Discussion

As a method for lesion localization using RFID technology, the use of endoscopic clips with attached RFID markers was evaluated. Endoscopic clips have previously been used for hemostasis and lesion marking in studies on lesion detection methods [9–12]. Park et al. found that using endoscopic clips to indicate the location of a lesion helped to determine an appropriate resection margin during surgery for early gastric cancer [13]. Hyung et al. and Kim et al. also introduced methods that used endoscopic metal clips to mark the location of a tumor and then detect the clips using laparoscopic ultrasonography or a portable abdominal radiograph [5, 6]. However, these methods require knowledge of ultrasonography and the assistance of a radiologist, and the accuracy of location detection can vary depending on the surgeon’s proficiency and the tumor’s location. Additionally, these methods can be time-consuming and may increase the total operation time, potentially affecting the patient’s postoperative recovery. Another method that utilizes real-time detection of the lesion by observing both the endoscopic and laparoscopic camera images has also been introduced [7]. However, this method also requires cooperation with the endoscopist and has yet to be established as a clinical standard.

Previous studies have demonstrated the use of RFID technology for lesion detection in order to improve the accuracy and efficiency of lesion localization, regardless of the surgeon’s experience and skill level [8]. The use of RFID tags as markers allows for intuitive detection through signals such as light or sound when the detector antenna is in close proximity to the RFID tag. This eliminates the need for expert knowledge or complex usage methods for lesion detection, and also reduces the time required for detection.

In order to ensure accurate marking, it is crucial that the marker is stably attached to the lesion. However, in some cases, the position of the clip may shift prior to surgery, leading to irritation [14]. Therefore, it is important to ensure that the clip is fixed securely to the tissue. To increase the clipping force of the clip, the angle of the jaw, or the end of the clip, was designed to be close to a vertical angle in accordance with endoscopic clip characteristics and guidelines [9, 10]. Additionally, the jaw was designed with a saw blade shape to enhance tissue engagement.

Since the clip is applied to the intestinal mucosa, it is important to use materials that are safe for the human body and have high acid resistance to withstand exposure to gastric acid. Furthermore, as the RFID system used for lesion detection operates through electromagnetic fields, it is necessary to use non-magnetic materials to avoid interference. Taking these factors into consideration, 304 and 316 types of stainless steel, commonly used in medical instruments, were chosen as materials for the clips used in the clinical experiments.

The feasibility of using stainless steel (STS) clips for lesion detection in endoscopic surgery was evaluated through computer-aided engineering (CAE) analysis and ex-vivo experimentation. The CAE analysis revealed that STS316L was not suitable for use as clips due to its poor shape retention and limited opening length, making it challenging to operate with the clip-fixing applicator. Conversely, clips made of STS304 were found to be suitable for use in ex-vivo experiments, where the results regarding tissue retention length and stability of the clips were obtained. The clips used in the ex-vivo experiments had thicknesses of 0.15 mm and 0.2 mm. Ex-vivo test results showed that the clip holding force and tissue retention length varied depending on the location where the clip was attached to the inner wall of the intestine. In-vivo experiments were performed to confirm the proper functioning of the RFID lesion detection system by attaching a clip for lesion detection to the inside of the porcine stomach. The location identified by the detector was marked with electrocautery, and it was confirmed that the clips were within the expected location after the gastrectomy. This demonstrates that the lesion detection system using the RFID system has high accuracy and clinical potential, but there are still challenges to its implementation in actual clinical practice.

In previous studies, resection margins for lesions in laparoscopic gastrectomy have been reported to be 2–3 cm from the expected location of the lesion in Korea [4], 2–5 cm according to the Japanese guidelines for gastric cancer treatment [15], and a minimum of 4 cm as recommended by the NCCN [16]. However, the length of the resection margin can be shortened by process of making the slide for pathologic result, and some of recent studies suggested that R0 resection is better important than absolute length of the margin [17–19]. Our study results showed that the maximum opening length of the prototype clip was 13 mm, and it could be attached at a distance of at least 6.5 mm from the lesion. Considering the error of the detected clip location in the in-vivo experiment, which was approximately 5 mm, the total error between the detected location and the lesion was expected to be around 11.5 mm. This suggests that the resection margin of around 10mm can be secured even if the stomach is resected close to the clip.

Since the safety margin is determined within a short distance, there might be concerns that common RFID products could affect lesion detection. However, common features like the NFC capability in smartphones or the EMV Contactless function used in credit cards operate at a carrier frequency of 13.56MHz, eliminating any potential for confusion. Generally, low-frequency RFID has a slower data transmission rate, making it unsuitable for everyday use. Thus, the likelihood of interference or confusion arising from an RFID tag operating in the 100KHz band is considerably low.

The time between diagnosis and surgery can often be prolonged, requiring the marking clip to remain in the body for several weeks. Literature indicates that endoscopic clips generally remain in the body for 18–26 days, though some cases have reported persistence for over a year [20]. Despite the proposed use of grasping forceps for clip removal [21], there remain challenges, such as bleeding and difficulties in removing the clip when it is fixed to the gastric wall. The current study was limited to in-vivo experiments, and further research is needed to evaluate the stability and removal of the clip in a clinical setting. Nonetheless, the stainless steel clips used in this study showed a low tissue retention capacity. To enhance the persistence of markers for use in patient-oriented clinical trials, further studies are needed to investigate ways to increase the clipping force, such as by incorporating higher strength materials or modifying clip arm shape.

In order to bring the RFID location detection system to clinical practice, there are several limitations that need to be addressed. One of the key challenges is to enhance the stability of the marking clip within the body for an extended duration, which can be achieved by experimenting with different materials or altering the design of the clip. Another important consideration is the safety issue related to the RFID chip and its method of attachment to the clip, which has not yet been fully addresses in this study.

## Conclusion

The study has evaluated the feasibility of using stainless steel (STS) clips for lesion detection in endoscopic surgery through pre-experimental computer-aided engineering (CAE) analysis and ex-vivo experimentation. The results of the CAE analysis revealed that STS304 was found to be suitable while STS316L was not. The ex-vivo experiments revealed that the clip holding force and tissue retention length varied depending on the location where the clip was attached to the inner wall of the intestine. In-vivo experiments were performed to confirm the proper functioning of the RFID lesion detection system by attaching a clip for lesion detection to the inside of the porcine stomach. The results showed high accuracy and clinical usefulness of the RFID lesion detection system. However, challenges still exist for its use in actual clinical field, such as ensuring the longevity of the clip and ensuring the safe attachment of the RFID tag. Further research is needed to address these challenges in order to use the RFID location detection system in actual clinical field.
