# Supplementary material for: Development and evaluation of RFID-integrated endoscopic clips for laparoscopic surgery marking
Source: PLoS One. 2024 May 2;19(5):e0302737. doi: 10.1371/journal.pone.0302737 (PMC11065250; doi:10.1371/journal.pone.0302737)
Supplement: Attachment — Submitted filename: Response to Reviewers.docx [file pone.0302737.s001.docx]

Reviewers' comments:

Comments to the Author

Reviewer #1:

--------------------------------------------------------------------------------------------------------------

Dear Reviewer and Editor,

I would like to express my deepest gratitude for the time and effort you have dedicated to the review of our manuscript. We have carefully considered each point raised during the review process and have undertaken thorough revisions accordingly. We firmly believe that the reviewer reports have allowed us to refine our arguments and bolster the validity and robustness of our research findings.

In the following sections, we have detailed how we addressed each comment, aiming to improve the overall quality and clarity of our work. And we presented the revised part of the manuscript in red color.

We hope that our revised manuscript meets the high standards of your esteemed journal and look forward to any additional comments or suggestions you may have.

Once again, we extend our sincere thanks for your invaluable contribution to improving our manuscript.

--------------------------------------------------------------------------------------------------------------

1. The authors show an interesting application of RFID tags for surgical applications.

There are some relevant measurement and simulation data on clip insertion force etc. However the draft doesn’t have almost any data on the RFID aspect. Even the name of the RFID component is not there (or I couldn’t find it easily). What measurements were done on the RFID positioning? Which locations, at what distance does it work? Which RF band is this? The RF energy will have a lot of absorption in tissue, so how was that mitigated?

**Authors’ response)**

- The authors conducted a pilot study to verify the clinical feasibility of RFID. We have previously reported on its characteristics, including detection range and the shape of the detection field. (Tumor localization using radio-frequency identification clip marker: experimental results of an ex vivo porcine model. Surgical Endoscopy 2019:33;1441–1450)
- In this study, our RFID component design was based on the EM4305 chipset (READELL EM4305, Xinyetong, China). This utilizes the ISO11784/5 protocol, and while the transmissible data is limited to less than 10 bytes, it demonstrates a high reliability in its performance within biological tissues.
- The RFID frequency band utilized is the 100KHz low-frequency band, which has demonstrated suitability within biological environments. While RF energy is indeed significantly absorbed by biological tissues, this characteristic is particularly pronounced at high-frequency bands (above 1MHz). In contrast, such limitations are minimally observed within the low-frequency domain. The measured detection range are distributed in ellipses with an average major axis of 12.5 mm and a minor axis of 5.3 mm. For further details on this, readers can refer to the 8th reference in this paper, previously published by the author.

2. No simulation on this aspect is available either. Where would the RF reader be placed and what sort of power it sends to the body? Is that within the SAR limit?

**Authors’ response)**

- Thank you for your insightful comment. As mentioned earlier, the RF reader was simulated ex-vivo in our previous pilot study. This research was conducted to check if it can be used in a situation similar to actual clinical setting.
- In this study, the RF reader is referred to as the tumor location detector and is developed in the form of a laparoscopic surgical instrument. This tumor location detector is designed as a long, slender rod, with an enamel coil at the tip for detecting RFID tags. It can be applied to a conventional laparoscopic 12mm trocar. Its impact on the Specific Absorption Rate (SAR) is negligible, owing to its low frequency, which results in low energy density. Furthermore, the detection energy is also less than 5 microwatts, making it unlikely to harm the human body.

3. Is the presence of the tag the only thing to be determined or the location is important too?

**Authors’ response)**

- This detection system was developed to localize the tumor location exactly. As described in the manuscript, there has been no effective and convenient way to identify tumor location during laparoscopic surgery. We previously reported that it could determine the location within an error of 10mm, which is clinically acceptable in view of oncological safety.
- As illustrated in the response to the first question, the detection distance is distributed in an elliptical shape. Through the strength of the signal returned by the RFID tag, inferring an approximate distance is also feasible. Theoretically, if two or more tumor location detectors are utilized, determining the position within a 2D plane is also achievable.

4. How long can the tag survive and what is the typical survival duration for this application?

**Authors’ response)**

- If the lesion area is marked with an RFID tag at the time of diagnosis and surgery is performed immediately, there would be no need for an additional endoscopy procedure for the surgery. It is a huge advantage in terms of reducing patient discomfort and being cost-effective. However, the time interval between diagnosis and surgery varies by region, hospital, and surgeon. The longer it can be maintained, the better, but permanent use is technically impossible. We assume that in clinical practice, a period of about four weeks would be an appropriate duration when utilizing this method. In cases where the time span between diagnosis and surgery exceeds four weeks, additional endoscopy before surgery is likely necessary for lesion localization.
- We performed experiments on persistence in an acidic environment, but since it isn't directly related to the main topic of this study, we didn't present the data in the main text. After immersing the clip tags in hydrochloric acid 3.5 pH and allowing them to sit for two weeks, performance testing revealed that 9 out of the 10 tags functioned normally. Based on these findings, it is inferred that their operational longevity can extend up to three weeks.

5. What about more than one tag in the vicinity? These days, people have RFID in all sorts of gadgets.

**Authors’ response)**

- Common features like the NFC capability in smartphones or the EMV Contactless function used in credit cards operate at a carrier frequency of 13.56MHz, eliminating any potential for confusion. Generally, low-frequency RFID has a slower data transmission rate, making it unsuitable for everyday use. Thus, the likelihood of interference or confusion arising from an RFID tag operating in the 100KHz band is considerably low.
- We added this in the discussion section of the manuscript.

6. What about the explantation of the tag? Is it necessary, how will that be done?

**Authors’ response)**

- Typically, the tag is removed alongside the tumor during the excision surgery following its installation. If the tumor removal surgery is delayed for any reason and exceeds the degradation timeline of the tag, there could be potential implications due to the corrosion on the affected organ. Nonetheless, it is highly likely to be naturally expelled from the body before this occurs.
