# Supplementary material for: Development and evaluation of RFID-integrated endoscopic clips for laparoscopic surgery marking
Source: PLoS One. 2024 May 2;19(5):e0302737. doi: 10.1371/journal.pone.0302737 (PMC11065250; doi:10.1371/journal.pone.0302737)
Supplement: Attachment — Submitted filename: Response to Reviewers.docx [file pone.0302737.s002.docx]

Reviewers' comments:

Comments to the Author

--------------------------------------------------------------------------------------------------------------

Dear Reviewer and Editor,

I would like to express my deepest gratitude for the time and effort you have dedicated to the review of our manuscript. We have carefully considered each point raised during the review process and have undertaken thorough revisions accordingly. We firmly believe that the reviewer reports have allowed us to refine our arguments and bolster the validity and robustness of our research findings.

In the following sections, we have detailed how we addressed each comment, aiming to improve the overall quality and clarity of our work. And we presented the revised part of the manuscript in red color.

We hope that our revised manuscript meets the high standards of your esteemed journal and look forward to any additional comments or suggestions you may have.

Once again, we extend our sincere thanks for your invaluable contribution to improving our manuscript.

--------------------------------------------------------------------------------------------------------------

Reviewer #2: The authors seem to have addressed most reviewers comments adequately.

For the Methods section, further details on the RFID chip and setup used are required. In particular, the reader details, chip model and make, and coil/capsule details along with dimensions should be added. Alternatively, detailed references should be made to help make the work reproducible.

Thank you for your comments. In this study, our RFID component design was based on the EM4305 chipset (EM4305, Xinyetong, China). This utilizes the ISO11784/5 protocol, and while the transmissible data is limited to less than 10 bytes, it demonstrates a high reliability in its performance within biological tissues. The RFID capsule has a diameter of 1.5 mm and a length of 7.5 mm. The reader used the EM4100 module, and the coil was fashioned into a cylindrical shape with a diameter of 8 mm and a height of 15 mm, set to 3.6 μH.

This content is added in Materials and methods - Materials section.

Reviewer #3: The authors present the design of a clip for laparoscopic surgery marking.

The novel contribution of the paper is not clear. The RFID module has been already presented by the author in previous research [1]. The use of endoscopic marking clips with integrated tags is not novel [2]. The authors designed a novel clip but it is unclear its difference from existing and commercial clips that justify a novel design. A comparison with existing clips in the experimental session is envisaged. In conclusion, the novelty of the contribution appears limited. Figure 2 overlaps Figure 1 in revision 1 and is of difficult readability.

Thank you for your comments. I completely agree with your opinion that the study could not be novel. However, this study was designed to evaluate the clinical feasibility and reproducibility in actual laparoscopic practice. Although we made the clip based on commercially available endoscopic clips, little is still known about how RFID tags function in the abdominal cavity. Because there are obvious differences between ex-vivo and in-vivo settings, the effectiveness of our integrated clips in locating lesions needs to be verified. In addition, to be compatible with endoscope equipment currently in clinical use, a clip with an RFID tag attached was designed based on the currently most commonly used endoscope clip. Although Wada et al. have reported the results of their IC chip-attached clip, our study reported more detailed results from ex-vivo and in-vivo experiments related to the clip manufacturing and actual detection process. Please consider these points for reviewing our study.

We have addressed this concern by relocating Figure 1 in the document.

[1] Joo, H. Y., Lee, B. E., Choi, C. I., Kim, D. H., Kim, G. H., Jeon, T. Y., ... & Ahn, S. (2019). Tumor localization using radio-frequency identification clip marker: experimental results of an ex vivo porcine model. Surgical Endoscopy, 33, 1441-1450.

[2] Wada, Y., Miyoshi, N., Ohue, M., Yasui, M., Fujino, S., Tomokuni, A., ... & Sakon, M. (2017). Endoscopic marking clip with an IC tag and receiving antenna to detect localization during laparoscopic surgery. Surgical Endoscopy, 31, 3056-3060.
