# Supplementary material for: Development and evaluation of RFID-integrated endoscopic clips for laparoscopic surgery marking
Source: PLoS One. 2024 May 2;19(5):e0302737. doi: 10.1371/journal.pone.0302737 (PMC11065250; doi:10.1371/journal.pone.0302737)
Supplement: Attachment — Submitted filename: Comments.docx [file pone.0302737.s003.docx]

Comments

The present study fails to introduce any significant novelty to the existing body of literature. Numerous analogous cases have been documented in prior research. However, there remains a critical need to thoroughly assess the feasibility and clinical applicability of the proposed design, particularly through in-vivo experimentation.

Thank you

Regards
