# Supplementary material for: Development and evaluation of RFID-integrated endoscopic clips for laparoscopic surgery marking
Source: PLoS One. 2024 May 2;19(5):e0302737. doi: 10.1371/journal.pone.0302737 (PMC11065250; doi:10.1371/journal.pone.0302737)
Supplement: Peer review history

# Peer review: Decision Letter 0

**DOI:** 10.1371/journal.pone.0302737.r001

**Authors:** Antonio Riveiro Rodríguez

7 Aug 2023

PONE-D-23-12315Development and Evaluation of RFID-integrated Endoscopic Clips for Laparoscopic Surgery MarkingPLOS ONE

Dear Dr. JOO,

Thank you for submitting your manuscript to PLOS ONE. After careful consideration, we feel that it has merit but does not fully meet PLOS ONE’s publication criteria as it currently stands. Therefore, we invite you to submit a revised version of the manuscript that addresses the points raised during the review process.

Please, address all the comments made by the reviewer.

Please submit your revised manuscript by Sep 21 2023 11:59PM. If you will need more time than this to complete your revisions, please reply to this message or contact the journal office at plosone@plos.org. When you're ready to submit your revision, log on to https://www.editorialmanager.com/pone/ and select the 'Submissions Needing Revision' folder to locate your manuscript file.

Please include the following items when submitting your revised manuscript:

- A rebuttal letter that responds to each point raised by the academic editor and reviewer(s). You should upload this letter as a separate file labeled 'Response to Reviewers'.
- A marked-up copy of your manuscript that highlights changes made to the original version. You should upload this as a separate file labeled 'Revised Manuscript with Track Changes'.
- An unmarked version of your revised paper without tracked changes. You should upload this as a separate file labeled 'Manuscript'.

If you would like to make changes to your financial disclosure, please include your updated statement in your cover letter. Guidelines for resubmitting your figure files are available below the reviewer comments at the end of this letter.

If applicable, we recommend that you deposit your laboratory protocols in protocols.io to enhance the reproducibility of your results. Protocols.io assigns your protocol its own identifier (DOI) so that it can be cited independently in the future. For instructions see: https://journals.plos.org/plosone/s/submission-guidelines#loc-laboratory-protocols. Additionally, PLOS ONE offers an option for publishing peer-reviewed Lab Protocol articles, which describe protocols hosted on protocols.io. Read more information on sharing protocols at https://plos.org/protocols?utm_medium=editorial-email&utm_source=authorletters&utm_campaign=protocols.

We look forward to receiving your revised manuscript.

Kind regards,

Antonio Riveiro Rodríguez, PhD

Academic Editor

PLOS ONE

Journal requirements:

When submitting your revision, we need you to address these additional requirements.

1. Please ensure that your manuscript meets PLOS ONE's style requirements, including those for file naming. The PLOS ONE style templates can be found at

https://journals.plos.org/plosone/s/file?id=wjVg/PLOSOne_formatting_sample_main_body.pdf and

https://journals.plos.org/plosone/s/file?id=ba62/PLOSOne_formatting_sample_title_authors_affiliations.pdf

2. To comply with PLOS ONE submissions requirements, in your Methods section, please provide additional information regarding the experiments involving animals and ensure you have included details on (1) methods of sacrifice, (2) methods of anesthesia and/or analgesia, and (3) efforts to alleviate suffering.

3. Please note that PLOS ONE has specific guidelines on code sharing for submissions in which author-generated code underpins the findings in the manuscript. In these cases, all author-generated code must be made available without restrictions upon publication of the work. Please review our guidelines at https://journals.plos.org/plosone/s/materials-and-software-sharing#loc-sharing-code and ensure that your code is shared in a way that follows best practice and facilitates reproducibility and reuse

4. Thank you for stating the following in the Acknowledgments Section of your manuscript:

"This work was financially supported by the Bio & Medical Technology Development Program of the NRF funded MSIP 2016M3A9E8942069 and 2017R1D1A1B03034853"

We note that you have provided additional information within the Acknowledgements Section that is not currently declared in your Funding Statement. Please note that funding information should not appear in the Acknowledgments section or other areas of your manuscript. We will only publish funding information present in the Funding Statement section of the online submission form.

Please remove any funding-related text from the manuscript and let us know how you would like to update your Funding Statement. Currently, your Funding Statement reads as follows:

"The funders had no role in study design, data collection and analysis, decision to publish, or preparation of the manuscript."

Please include your amended statements within your cover letter; we will change the online submission form on your behalf.

5. We note that the grant information you provided in the ‘Funding Information’ and ‘Financial Disclosure’ sections do not match.

When you resubmit, please ensure that you provide the correct grant numbers for the awards you received for your study in the ‘Funding Information’ section.

6. Thank you for stating the following financial disclosure:

"The funders had no role in study design, data collection and analysis, decision to publish, or preparation of the manuscript."

At this time, please address the following queries:

a)        Please clarify the sources of funding (financial or material support) for your study. List the grants or organizations that supported your study, including funding received from your institution.

b)        State what role the funders took in the study. If the funders had no role in your study, please state: “The funders had no role in study design, data collection and analysis, decision to publish, or preparation of the manuscript.”

c)        If any authors received a salary from any of your funders, please state which authors and which funders.

d)        If you did not receive any funding for this study, please state: “The authors received no specific funding for this work.”

Please include your amended statements within your cover letter; we will change the online submission form on your behalf.

7. Please include your full ethics statement in the ‘Methods’ section of your manuscript file. In your statement, please include the full name of the IRB or ethics committee who approved or waived your study, as well as whether or not you obtained informed written or verbal consent. If consent was waived for your study, please include this information in your statement as well.

Reviewers' comments:

Reviewer's Responses to Questions

**Comments to the Author**

1. Is the manuscript technically sound, and do the data support the conclusions?

The manuscript must describe a technically sound piece of scientific research with data that supports the conclusions. Experiments must have been conducted rigorously, with appropriate controls, replication, and sample sizes. The conclusions must be drawn appropriately based on the data presented. 

Reviewer #1: No

**********

2. Has the statistical analysis been performed appropriately and rigorously? 

Reviewer #1: Yes

**********

3. Have the authors made all data underlying the findings in their manuscript fully available?

The PLOS Data policy requires authors to make all data underlying the findings described in their manuscript fully available without restriction, with rare exception (please refer to the Data Availability Statement in the manuscript PDF file). The data should be provided as part of the manuscript or its supporting information, or deposited to a public repository. For example, in addition to summary statistics, the data points behind means, medians and variance measures should be available. If there are restrictions on publicly sharing data—e.g. participant privacy or use of data from a third party—those must be specified.

Reviewer #1: No

**********

4. Is the manuscript presented in an intelligible fashion and written in standard English?

PLOS ONE does not copyedit accepted manuscripts, so the language in submitted articles must be clear, correct, and unambiguous. Any typographical or grammatical errors should be corrected at revision, so please note any specific errors here.

Reviewer #1: Yes

**********

5. Review Comments to the Author

Please use the space provided to explain your answers to the questions above. You may also include additional comments for the author, including concerns about dual publication, research ethics, or publication ethics. (Please upload your review as an attachment if it exceeds 20,000 characters)

Reviewer #1: The authors show an interesting application of RFID tags for surgical applications.

There are some relevant measurement and simulation data on clip insertion force etc. However the draft doesn’t have almost any data on the RFID aspect. Even the name of the RFID component is not there (or I couldn’t find it easily). What measurements were done on the RFID positioning? Which locations, at what distance does it work? Which RF band is this? The RF energy will have a lot of absorption in tissue, so how was that mitigated?

No simulation on this aspect is available either. Where would the RF reader be placed and what sort of power it sends to the body? Is that within the SAR limit?

Is the presence of the tag the only thing to be determined or the location is important too?

How long can the tag survive and what is the typical survival duration for this application?

What about more than one tag in the vicinity? These days, people have RFID in all sorts of gadgets.

What about the explantation of the tag? Is it necessary, how will that be done?

**********

6. PLOS authors have the option to publish the peer review history of their article (what does this mean?). If published, this will include your full peer review and any attached files.

If you choose “no”, your identity will remain anonymous but your review may still be made public.

**Do you want your identity to be public for this peer review?** For information about this choice, including consent withdrawal, please see our Privacy Policy.

Reviewer #1: No

**********

[NOTE: If reviewer comments were submitted as an attachment file, they will be attached to this email and accessible via the submission site. Please log into your account, locate the manuscript record, and check for the action link "View Attachments". If this link does not appear, there are no attachment files.]

While revising your submission, please upload your figure files to the Preflight Analysis and Conversion Engine (PACE) digital diagnostic tool, https://pacev2.apexcovantage.com/. PACE helps ensure that figures meet PLOS requirements. To use PACE, you must first register as a user. Registration is free. Then, login and navigate to the UPLOAD tab, where you will find detailed instructions on how to use the tool. If you encounter any issues or have any questions when using PACE, please email PLOS at figures@plos.org. Please note that Supporting Information files do not need this step.

# Author comment: Author response to Decision Letter 0

**DOI:** 10.1371/journal.pone.0302737.r002

8 Oct 2023

Reviewers' comments:

Comments to the Author

Reviewer #1: 

--------------------------------------------------------------------------------------------------------------

Dear Reviewer and Editor,

 I would like to express my deepest gratitude for the time and effort you have dedicated to the review of our manuscript. We have carefully considered each point raised during the review process and have undertaken thorough revisions accordingly. We firmly believe that the reviewer reports have allowed us to refine our arguments and bolster the validity and robustness of our research findings.

 In the following sections, we have detailed how we addressed each comment, aiming to improve the overall quality and clarity of our work. And we presented the revised part of the manuscript in red color.

 We hope that our revised manuscript meets the high standards of your esteemed journal and look forward to any additional comments or suggestions you may have.

 Once again, we extend our sincere thanks for your invaluable contribution to improving our manuscript.

--------------------------------------------------------------------------------------------------------------

1. The authors show an interesting application of RFID tags for surgical applications.

There are some relevant measurement and simulation data on clip insertion force etc. However the draft doesn’t have almost any data on the RFID aspect. Even the name of the RFID component is not there (or I couldn’t find it easily). What measurements were done on the RFID positioning? Which locations, at what distance does it work? Which RF band is this? The RF energy will have a lot of absorption in tissue, so how was that mitigated?

Authors’ response)

- The authors conducted a pilot study to verify the clinical feasibility of RFID. We have previously reported on its characteristics, including detection range and the shape of the detection field. (Tumor localization using radio-frequency identification clip marker: experimental results of an ex vivo porcine model. Surgical Endoscopy 2019:33;1441–1450)

- In this study, our RFID component design was based on the EM4305 chipset (READELL EM4305, Xinyetong, China). This utilizes the ISO11784/5 protocol, and while the transmissible data is limited to less than 10 bytes, it demonstrates a high reliability in its performance within biological tissues.

- The RFID frequency band utilized is the 100KHz low-frequency band, which has demonstrated suitability within biological environments. While RF energy is indeed significantly absorbed by biological tissues, this characteristic is particularly pronounced at high-frequency bands (above 1MHz). In contrast, such limitations are minimally observed within the low-frequency domain. The measured detection range are distributed in ellipses with an average major axis of 12.5 mm and a minor axis of 5.3 mm. For further details on this, readers can refer to the 8th reference in this paper, previously published by the author.

2. No simulation on this aspect is available either. Where would the RF reader be placed and what sort of power it sends to the body? Is that within the SAR limit?

Authors’ response)

- Thank you for your insightful comment. As mentioned earlier, the RF reader was simulated ex-vivo in our previous pilot study. This research was conducted to check if it can be used in a situation similar to actual clinical setting.

- In this study, the RF reader is referred to as the tumor location detector and is developed in the form of a laparoscopic surgical instrument. This tumor location detector is designed as a long, slender rod, with an enamel coil at the tip for detecting RFID tags. It can be applied to a conventional laparoscopic 12mm trocar. Its impact on the Specific Absorption Rate (SAR) is negligible, owing to its low frequency, which results in low energy density. Furthermore, the detection energy is also less than 5 microwatts, making it unlikely to harm the human body.

3. Is the presence of the tag the only thing to be determined or the location is important too?

Authors’ response)

- This detection system was developed to localize the tumor location exactly. As described in the manuscript, there has been no effective and convenient way to identify tumor location during laparoscopic surgery. We previously reported that it could determine the location within an error of 10mm, which is clinically acceptable in view of oncological safety.

- As illustrated in the response to the first question, the detection distance is distributed in an elliptical shape. Through the strength of the signal returned by the RFID tag, inferring an approximate distance is also feasible. Theoretically, if two or more tumor location detectors are utilized, determining the position within a 2D plane is also achievable.

4. How long can the tag survive and what is the typical survival duration for this application?

Authors’ response)

- If the lesion area is marked with an RFID tag at the time of diagnosis and surgery is performed immediately, there would be no need for an additional endoscopy procedure for the surgery. It is a huge advantage in terms of reducing patient discomfort and being cost-effective. However, the time interval between diagnosis and surgery varies by region, hospital, and surgeon. The longer it can be maintained, the better, but permanent use is technically impossible. We assume that in clinical practice, a period of about four weeks would be an appropriate duration when utilizing this method. In cases where the time span between diagnosis and surgery exceeds four weeks, additional endoscopy before surgery is likely necessary for lesion localization.

- We performed experiments on persistence in an acidic environment, but since it isn't directly related to the main topic of this study, we didn't present the data in the main text. After immersing the clip tags in hydrochloric acid 3.5 pH and allowing them to sit for two weeks, performance testing revealed that 9 out of the 10 tags functioned normally. Based on these findings, it is inferred that their operational longevity can extend up to three weeks.

5. What about more than one tag in the vicinity? These days, people have RFID in all sorts of gadgets.

Authors’ response)

- Common features like the NFC capability in smartphones or the EMV Contactless function used in credit cards operate at a carrier frequency of 13.56MHz, eliminating any potential for confusion. Generally, low-frequency RFID has a slower data transmission rate, making it unsuitable for everyday use. Thus, the likelihood of interference or confusion arising from an RFID tag operating in the 100KHz band is considerably low.

- We added this in the discussion section of the manuscript.

6. What about the explantation of the tag? Is it necessary, how will that be done?

Authors’ response)

- Typically, the tag is removed alongside the tumor during the excision surgery following its installation. If the tumor removal surgery is delayed for any reason and exceeds the degradation timeline of the tag, there could be potential implications due to the corrosion on the affected organ. Nonetheless, it is highly likely to be naturally expelled from the body before this occurs.

Attachment Submitted filename: Response to Reviewers.docx

# Peer review: Decision Letter 1

**DOI:** 10.1371/journal.pone.0302737.r003

**Authors:** Antonio Riveiro Rodríguez

19 Dec 2023

PONE-D-23-12315R1Development and Evaluation of RFID-integrated Endoscopic Clips for Laparoscopic Surgery MarkingPLOS ONE

Dear Dr. JOO,

Thank you for submitting your manuscript to PLOS ONE. After careful consideration, we feel that it has merit but does not fully meet PLOS ONE’s publication criteria as it currently stands. Therefore, we invite you to submit a revised version of the manuscript that addresses the points raised during the review process.

 Please, address all the comments made by the reviewers.

Please submit your revised manuscript by Feb 02 2024 11:59PM. If you will need more time than this to complete your revisions, please reply to this message or contact the journal office at plosone@plos.org. When you're ready to submit your revision, log on to https://www.editorialmanager.com/pone/ and select the 'Submissions Needing Revision' folder to locate your manuscript file.

Please include the following items when submitting your revised manuscript:

- A rebuttal letter that responds to each point raised by the academic editor and reviewer(s). You should upload this letter as a separate file labeled 'Response to Reviewers'.
- A marked-up copy of your manuscript that highlights changes made to the original version. You should upload this as a separate file labeled 'Revised Manuscript with Track Changes'.
- An unmarked version of your revised paper without tracked changes. You should upload this as a separate file labeled 'Manuscript'.

If you would like to make changes to your financial disclosure, please include your updated statement in your cover letter. Guidelines for resubmitting your figure files are available below the reviewer comments at the end of this letter.

If applicable, we recommend that you deposit your laboratory protocols in protocols.io to enhance the reproducibility of your results. Protocols.io assigns your protocol its own identifier (DOI) so that it can be cited independently in the future. For instructions see: https://journals.plos.org/plosone/s/submission-guidelines#loc-laboratory-protocols. Additionally, PLOS ONE offers an option for publishing peer-reviewed Lab Protocol articles, which describe protocols hosted on protocols.io. Read more information on sharing protocols at https://plos.org/protocols?utm_medium=editorial-email&utm_source=authorletters&utm_campaign=protocols.

We look forward to receiving your revised manuscript.

Kind regards,

Antonio Riveiro Rodríguez, PhD

Academic Editor

PLOS ONE

Reviewers' comments:

Reviewer's Responses to Questions

**Comments to the Author**

1. If the authors have adequately addressed your comments raised in a previous round of review and you feel that this manuscript is now acceptable for publication, you may indicate that here to bypass the “Comments to the Author” section, enter your conflict of interest statement in the “Confidential to Editor” section, and submit your "Accept" recommendation.

Reviewer #2: All comments have been addressed

Reviewer #3: (No Response)

**********

2. Is the manuscript technically sound, and do the data support the conclusions?

The manuscript must describe a technically sound piece of scientific research with data that supports the conclusions. Experiments must have been conducted rigorously, with appropriate controls, replication, and sample sizes. The conclusions must be drawn appropriately based on the data presented. 

Reviewer #2: Yes

Reviewer #3: Partly

**********

3. Has the statistical analysis been performed appropriately and rigorously? 

Reviewer #2: Yes

Reviewer #3: N/A

**********

4. Have the authors made all data underlying the findings in their manuscript fully available?

The PLOS Data policy requires authors to make all data underlying the findings described in their manuscript fully available without restriction, with rare exception (please refer to the Data Availability Statement in the manuscript PDF file). The data should be provided as part of the manuscript or its supporting information, or deposited to a public repository. For example, in addition to summary statistics, the data points behind means, medians and variance measures should be available. If there are restrictions on publicly sharing data—e.g. participant privacy or use of data from a third party—those must be specified.

Reviewer #2: Yes

Reviewer #3: No

**********

5. Is the manuscript presented in an intelligible fashion and written in standard English?

PLOS ONE does not copyedit accepted manuscripts, so the language in submitted articles must be clear, correct, and unambiguous. Any typographical or grammatical errors should be corrected at revision, so please note any specific errors here.

Reviewer #2: Yes

Reviewer #3: Yes

**********

6. Review Comments to the Author

Please use the space provided to explain your answers to the questions above. You may also include additional comments for the author, including concerns about dual publication, research ethics, or publication ethics. (Please upload your review as an attachment if it exceeds 20,000 characters)

Reviewer #2: The authors seem to have addressed most reviewers comments adequately.

For the Methods section, further details on the RFID chip and setup used are required. In particular, the reader details, chip model and make, and coil/capsule details along with dimensions should be added. Alternatively, detailed references should be made to help make the work reproducible.

Reviewer #3: The authors present the design of a clip for laparoscopic surgery marking.

The novel contribution of the paper is not clear. The RFID module has been already presented by the author in previous research [1]. The use of endoscopic marking clips with integrated tags is not novel [2]. The authors designed a novel clip but it is unclear its difference from existing and commercial clips that justify a novel design. A comparison with existing clips in the experimental session is envisaged. In conclusion, the novelty of the contribution appears limited. Figure 2 overlaps Figure 1 in revision 1 and is of difficult readability.

[1] Joo, H. Y., Lee, B. E., Choi, C. I., Kim, D. H., Kim, G. H., Jeon, T. Y., ... & Ahn, S. (2019). Tumor localization using radio-frequency identification clip marker: experimental results of an ex vivo porcine model. Surgical Endoscopy, 33, 1441-1450.

[2] Wada, Y., Miyoshi, N., Ohue, M., Yasui, M., Fujino, S., Tomokuni, A., ... & Sakon, M. (2017). Endoscopic marking clip with an IC tag and receiving antenna to detect localization during laparoscopic surgery. Surgical Endoscopy, 31, 3056-3060.

**********

7. PLOS authors have the option to publish the peer review history of their article (what does this mean?). If published, this will include your full peer review and any attached files.

If you choose “no”, your identity will remain anonymous but your review may still be made public.

**Do you want your identity to be public for this peer review?** For information about this choice, including consent withdrawal, please see our Privacy Policy.

Reviewer #2: No

Reviewer #3: No

**********

[NOTE: If reviewer comments were submitted as an attachment file, they will be attached to this email and accessible via the submission site. Please log into your account, locate the manuscript record, and check for the action link "View Attachments". If this link does not appear, there are no attachment files.]

While revising your submission, please upload your figure files to the Preflight Analysis and Conversion Engine (PACE) digital diagnostic tool, https://pacev2.apexcovantage.com/. PACE helps ensure that figures meet PLOS requirements. To use PACE, you must first register as a user. Registration is free. Then, login and navigate to the UPLOAD tab, where you will find detailed instructions on how to use the tool. If you encounter any issues or have any questions when using PACE, please email PLOS at figures@plos.org. Please note that Supporting Information files do not need this step.

# Author comment: Author response to Decision Letter 1

**DOI:** 10.1371/journal.pone.0302737.r004

16 Feb 2024

Reviewers' comments:

Comments to the Author

--------------------------------------------------------------------------------------------------------------

Dear Reviewer and Editor,

 I would like to express my deepest gratitude for the time and effort you have dedicated to the review of our manuscript. We have carefully considered each point raised during the review process and have undertaken thorough revisions accordingly. We firmly believe that the reviewer reports have allowed us to refine our arguments and bolster the validity and robustness of our research findings.

 In the following sections, we have detailed how we addressed each comment, aiming to improve the overall quality and clarity of our work. And we presented the revised part of the manuscript in red color.

 We hope that our revised manuscript meets the high standards of your esteemed journal and look forward to any additional comments or suggestions you may have.

 Once again, we extend our sincere thanks for your invaluable contribution to improving our manuscript.

--------------------------------------------------------------------------------------------------------------

Reviewer #2: The authors seem to have addressed most reviewers comments adequately.

For the Methods section, further details on the RFID chip and setup used are required. In particular, the reader details, chip model and make, and coil/capsule details along with dimensions should be added. Alternatively, detailed references should be made to help make the work reproducible.

Thank you for your comments. In this study, our RFID component design was based on the EM4305 chipset (EM4305, Xinyetong, China). This utilizes the ISO11784/5 protocol, and while the transmissible data is limited to less than 10 bytes, it demonstrates a high reliability in its performance within biological tissues. The RFID capsule has a diameter of 1.5 mm and a length of 7.5 mm. The reader used the EM4100 module, and the coil was fashioned into a cylindrical shape with a diameter of 8 mm and a height of 15 mm, set to 3.6 μH.

This content is added in Materials and methods - Materials section.

Reviewer #3: The authors present the design of a clip for laparoscopic surgery marking.

The novel contribution of the paper is not clear. The RFID module has been already presented by the author in previous research [1]. The use of endoscopic marking clips with integrated tags is not novel [2]. The authors designed a novel clip but it is unclear its difference from existing and commercial clips that justify a novel design. A comparison with existing clips in the experimental session is envisaged. In conclusion, the novelty of the contribution appears limited. Figure 2 overlaps Figure 1 in revision 1 and is of difficult readability.

Thank you for your comments. I completely agree with your opinion that the study could not be novel. However, this study was designed to evaluate the clinical feasibility and reproducibility in actual laparoscopic practice. Although we made the clip based on commercially available endoscopic clips, little is still known about how RFID tags function in the abdominal cavity. Because there are obvious differences between ex-vivo and in-vivo settings, the effectiveness of our integrated clips in locating lesions needs to be verified. In addition, to be compatible with endoscope equipment currently in clinical use, a clip with an RFID tag attached was designed based on the currently most commonly used endoscope clip. Although Wada et al. have reported the results of their IC chip-attached clip, our study reported more detailed results from ex-vivo and in-vivo experiments related to the clip manufacturing and actual detection process. Please consider these points for reviewing our study.

We have addressed this concern by relocating Figure 1 in the document.

[1] Joo, H. Y., Lee, B. E., Choi, C. I., Kim, D. H., Kim, G. H., Jeon, T. Y., ... & Ahn, S. (2019). Tumor localization using radio-frequency identification clip marker: experimental results of an ex vivo porcine model. Surgical Endoscopy, 33, 1441-1450.

[2] Wada, Y., Miyoshi, N., Ohue, M., Yasui, M., Fujino, S., Tomokuni, A., ... & Sakon, M. (2017). Endoscopic marking clip with an IC tag and receiving antenna to detect localization during laparoscopic surgery. Surgical Endoscopy, 31, 3056-3060.

Attachment Submitted filename: Response to Reviewers.docx

# Peer review: Decision Letter 2

**DOI:** 10.1371/journal.pone.0302737.r005

**Authors:** Antonio Riveiro Rodríguez

11 Apr 2024

Development and Evaluation of RFID-integrated Endoscopic Clips for Laparoscopic Surgery Marking

PONE-D-23-12315R2

Dear Dr. JOO,

We’re pleased to inform you that your manuscript has been judged scientifically suitable for publication and will be formally accepted for publication once it meets all outstanding technical requirements.

Within one week, you’ll receive an e-mail detailing the required amendments. When these have been addressed, you’ll receive a formal acceptance letter and your manuscript will be scheduled for publication.

An invoice will be generated when your article is formally accepted. Please note, if your institution has a publishing partnership with PLOS and your article meets the relevant criteria, all or part of your publication costs will be covered. Please make sure your user information is up-to-date by logging into Editorial Manager at Editorial Manager® and clicking the ‘Update My Information' link at the top of the page. If you have any questions relating to publication charges, please contact our Author Billing department directly at authorbilling@plos.org.

If your institution or institutions have a press office, please notify them about your upcoming paper to help maximize its impact. If they’ll be preparing press materials, please inform our press team as soon as possible -- no later than 48 hours after receiving the formal acceptance. Your manuscript will remain under strict press embargo until 2 pm Eastern Time on the date of publication. For more information, please contact onepress@plos.org.

Kind regards,

Antonio Riveiro Rodríguez, PhD

Academic Editor

PLOS ONE

Additional Editor Comments (optional):

Reviewers' comments:

Reviewer's Responses to Questions

**Comments to the Author**

1. If the authors have adequately addressed your comments raised in a previous round of review and you feel that this manuscript is now acceptable for publication, you may indicate that here to bypass the “Comments to the Author” section, enter your conflict of interest statement in the “Confidential to Editor” section, and submit your "Accept" recommendation.

Reviewer #4: All comments have been addressed

**********

2. Is the manuscript technically sound, and do the data support the conclusions?

The manuscript must describe a technically sound piece of scientific research with data that supports the conclusions. Experiments must have been conducted rigorously, with appropriate controls, replication, and sample sizes. The conclusions must be drawn appropriately based on the data presented. 

Reviewer #4: Yes

**********

3. Has the statistical analysis been performed appropriately and rigorously? 

Reviewer #4: Yes

**********

4. Have the authors made all data underlying the findings in their manuscript fully available?

The PLOS Data policy requires authors to make all data underlying the findings described in their manuscript fully available without restriction, with rare exception (please refer to the Data Availability Statement in the manuscript PDF file). The data should be provided as part of the manuscript or its supporting information, or deposited to a public repository. For example, in addition to summary statistics, the data points behind means, medians and variance measures should be available. If there are restrictions on publicly sharing data—e.g. participant privacy or use of data from a third party—those must be specified.

Reviewer #4: Yes

**********

5. Is the manuscript presented in an intelligible fashion and written in standard English?

PLOS ONE does not copyedit accepted manuscripts, so the language in submitted articles must be clear, correct, and unambiguous. Any typographical or grammatical errors should be corrected at revision, so please note any specific errors here.

Reviewer #4: Yes

**********

6. Review Comments to the Author

Please use the space provided to explain your answers to the questions above. You may also include additional comments for the author, including concerns about dual publication, research ethics, or publication ethics. (Please upload your review as an attachment if it exceeds 20,000 characters)

Reviewer #4: The present study fails to introduce any significant novelty to the existing body of literature. Numerous analogous cases have been documented in prior research. However, there remains a critical need to thoroughly assess the feasibility and clinical applicability of the proposed design, particularly through in-vivo experimentation.

**********

7. PLOS authors have the option to publish the peer review history of their article (what does this mean?). If published, this will include your full peer review and any attached files.

If you choose “no”, your identity will remain anonymous but your review may still be made public.

**Do you want your identity to be public for this peer review?** For information about this choice, including consent withdrawal, please see our Privacy Policy.

Reviewer #4: **Yes: **Diwakar Shankar

**********

Attachment Submitted filename: Comments.docx

# Editor report: Acceptance letter

**DOI:** 10.1371/journal.pone.0302737.r006

**Authors:** Antonio Riveiro Rodríguez

24 Apr 2024

PONE-D-23-12315R2 

PLOS ONE

Dear Dr. Joo, 

I'm pleased to inform you that your manuscript has been deemed suitable for publication in PLOS ONE. Congratulations! Your manuscript is now being handed over to our production team.

At this stage, our production department will prepare your paper for publication. This includes ensuring the following:

* All references, tables, and figures are properly cited

* All relevant supporting information is included in the manuscript submission,

* There are no issues that prevent the paper from being properly typeset

If revisions are needed, the production department will contact you directly to resolve them. If no revisions are needed, you will receive an email when the publication date has been set. At this time, we do not offer pre-publication proofs to authors during production of the accepted work. Please keep in mind that we are working through a large volume of accepted articles, so please give us a few weeks to review your paper and let you know the next and final steps. 

Lastly, if your institution or institutions have a press office, please let them know about your upcoming paper now to help maximize its impact. If they'll be preparing press materials, please inform our press team within the next 48 hours. Your manuscript will remain under strict press embargo until 2 pm Eastern Time on the date of publication. For more information, please contact onepress@plos.org.

If we can help with anything else, please email us at customercare@plos.org.

Thank you for submitting your work to PLOS ONE and supporting open access. 

Kind regards, 

PLOS ONE Editorial Office Staff

on behalf of

Dr. Antonio Riveiro Rodríguez 

Academic Editor

PLOS ONE
